# Skeletal Muscle Pathology in Autosomal Recessive Cerebellar Ataxias: Insights from Marinesco–Sjögren Syndrome

**DOI:** 10.3390/ijms26146736

**Published:** 2025-07-14

**Authors:** Fabio Bellia, Luca Federici, Valentina Gatta, Giuseppe Calabrese, Michele Sallese

**Affiliations:** 1 Department of Innovative Technologies in Medicine and Dentistry, “G. d’Annunzio” University of Chieti-Pescara, 66100 Chieti, Italy; fabio.bellia@unich.it (F.B.); lfederici@unich.it (L.F.); 2 Center for Advanced Studies and Technology (CAST), “G. d’Annunzio” University of Chieti-Pescara, 66100 Chieti, Italy; 3 Department of Psychological, Health and Territorial Sciences, School of Medicine and Health Sciences, “G. d’Annunzio” University of Chieti-Pescara, 66100 Chieti, Italy; v.gatta@unich.it; 4 Functional Genetics Unit, Center of Excellence on Aging (Ce.S.I.-MeT), 66100 Chieti, Italy; 5 Department of Oncology Hematology, Pescara Hospital, 65124 Pescara, Italy; giuseppe.calabrese@unich.it; 6 Department of Medical, Oral and Biotechnological Sciences, “G. d’Annunzio” University of Chieti-Pescara, 66100 Pescara, Italy

**Keywords:** cerebellar ataxias, skeletal muscle, preclinical models, muscle biopsy

## Abstract

Cerebellar ataxias are a group of disorders characterized by clumsy movements because of defective muscle control. In affected individuals, muscular impairment might have an impact on activities like walking, balance, hand coordination, speech, and feeding, as well as eye movements. The development of symptoms typically takes place during the span of adolescence, and it has the potential to cause distress for individuals in many areas of their lives, including professional and interpersonal relationships. Although skeletal muscle is understudied in ataxias, its examination may provide hitherto unexplored details in this family of disorders. Observing muscle involvement can assist in diagnosing conditions where genetic tests alone are inconclusive. Furthermore, it helps determine the stage of progression of a pathology that might otherwise be challenging to assess. In this study, we reviewed the main scientific literature reporting on skeletal muscle examination in autosomal recessive cerebellar ataxias (ARCAs), with a focus on the rare Marinesco–Sjögren syndrome. (MSS). Our aim was to highlight the similarities in muscle alterations observed in ARCA patients while also considering data gathered from preclinical models. Analyzing the similarities among these disorders could enhance our understanding of the unidentified mechanisms underlying the phenotypic evolution of some less common conditions.

## 1. Introduction to ARCAs

Ataxias are usually defined as a loss of motor coordination of a pathological nature, manifesting as a neurological disease or as part of a multisystemic disorder. Most of this disease class involves the dysfunction of cerebellar neurons, other than their afferent and efferent connections. Inherited cerebellar ataxias can be divided into four main classes: autosomal recessive (ARCA), autosomal dominant (ADCA), X-linked, and mitochondrial cerebellar ataxias, all of which are caused by dysfunction and degeneration of the cerebellar cortex [1]. A common feature of these disorders is the alteration of the cerebellum, which typically occurs before the age of 20. Additionally, abnormalities affecting peripheral tissues highlight some heterogeneity. Inherited cerebellar ataxias have varying effects on the skeletal muscle regions of affected individuals, resulting from either the direct consequences of cerebellar ataxias or a parallel process of brain degeneration.

From a biological point of view, the various ARCAs can be classified as follows: ataxias with impaired DNA repair mechanism, such as telangiectasia (A–T) and oculomotor apraxia (AOA) 1 and 2; degenerative ataxias, such as Friedreich ataxia (FRDA), Coenzyme Q10 deficiency (CoQ10), mitochondrial recessive ataxic syndrome (such as the spastic paraplegic 7, SPG7), autosomal recessive ataxia type 1 (ARCA-1), also known as spinocerebellar ataxia type 8 (SCAR8), and the Marinesco–Sjögren syndrome (MSS); congenital ataxias, such as Joubert syndromes and Cayman ataxia; metabolic ataxias, such as vitamin E deficiency, Refsum’s, and Wilson’s disease. Changes in various molecular pathways, including oxidative stress, inefficient DNA repair, mitochondrial malfunction, protein misfolding, and aberrant cytoskeletal proteins, are the mechanisms that cause an improperly developed cerebellum in this class of disorders [2].

The following paragraphs will briefly discuss the main features of the various ARCAs, with an emphasis on how they differ from and are similar to Marinesco–Sjögren’s syndrome. We will also discuss the muscular aspects and how the various ataxias differ from one other.

## 2. Overview of Marinesco–Sjögren Syndrome

In 1931, Marinesco and colleagues published the first description of MSS, a syndrome characterized by congenital cataracts, cerebellar ataxia, and mental and physical impairment [3]. Subsequent research by Sjögren and colleagues confirmed the genetic basis of this syndrome and established its autosomal recessive inheritance [4]. Nowadays, MSS is considered a multisystemic condition that affects approximately 1–9 out of 1,000,000 individuals and has a variety of phenotypes, although it has certain “cardinal” traits [5]. Contrary to other movement disorders, such as Parkinson’s and Huntington’s disease [6,7,8], gender preference has not been documented in the literature. Unlike other ARCAs, such as FRDA, individuals with MSS appear to have a normal life span [9].

**Figure 1:**
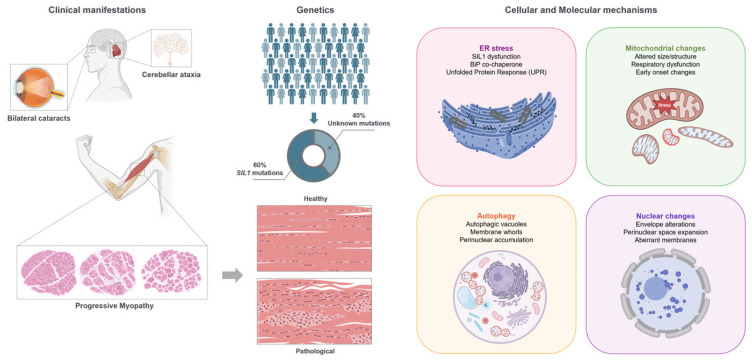
MSS characteristics. The figure represents the main clinical features observed in MSS patients. On the right side, the major observations in muscle biopsies are schematically represented.

Cerebellar ataxia, bilateral cataracts, and progressive myopathy are the three “cardinal” features of MSS patients (Figure 1). These three symptoms occur in MSS chronologically [10] and are the main characteristics utilized for a clinical diagnosis. Cerebellar ataxia develops after the first year of life. It is caused by severe degeneration of the cerebellar lobules due to granule cell loss and Purkinje cells (PC) degeneration [11]. Along with cerebellar impairment, affected individuals often exhibit intellectual disability, a condition characterized by difficulties in learning new things and regressive tendencies resulting from persistent nerve cell atrophy [3,5]. Bilateral cataracts typically coexist with cerebellar degeneration; however, unlike cerebellar impairment, the precise mechanisms of eye involvement in this condition remain unclear. In addition, patients with MSS exhibit myopathy and skeletal muscle degeneration. Analysis of muscle tissue by light and electron microscopy identified several differences among patients (Supplementary Table S1). In general, MSS muscles exhibit high variability in myofiber size and morphology, degradation of muscular fibers with the replacement of connective and fatty tissue, internalization of myonuclei, and aberrant membrane structures surrounding the nuclei. In addition, the autophagy revealed in cells from MSS patients also suggested potential metabolic abnormalities in muscle cells [12].

### 2.1. Genetic and Molecular Basis of MSS

The *SIL1* gene is involved upstream in most cases of MSS, with loss-of-function mutations producing altered forms of the protein [10]. The *SIL1* gene, located on chromosome 5q31, encodes a nucleotide exchange factor (NEF) that serves as a co-chaperone for the endoplasmic reticulum (ER) chaperone immunoglobulin binding protein (BiP), also referred to as glucose-regulated protein of 78 kDa (GPR78). ER-resident NEFs, such as SIL1 and the 150 kDa oxygen-regulated protein (ORP150; also known as GRP170), regulate the protein folding ability of BiP by releasing ADP from the BiP-client protein complex, thus allowing a fresh ATP molecule to bind [13]. MSS is recognized as a disorder of the ER, as the absence of functional SIL1 affects normal BiP functions, including its roles in protein folding and control of ER stress [14]. Under physiological circumstances, BiP associates with transmembrane UPR sensors PERK, IRE1, and ATF6. In response to ER stress, BiP dissociates from PERK, IRE1, and ATF6, allowing these sensors to become activated, often through phosphorylation [15,16]. ER stress acts as a protective mechanism to manage the accumulation of misfolded proteins within the ER lumen, and elevated ER stress markers have been observed in patients with MSS [13,17].

Several variations in *SIL1* have been identified, including premature truncations, frame-shift mutations, in-frame deletions, and missense mutations [18,19]. According to Howes and colleagues [20], all of these possible gene mutations result in a truncated or C-terminal modified protein, which impairs SIL1 protein function and leads to an accumulation of unfolded proteins within the cell, eventually triggering the unfolded protein response (UPR). Nonetheless, even among those who exhibit all the syndrome’s hallmarks, 40% of MSS patients do not have any *SIL1* mutation. To date, the other genes responsible for MSS development remain unclear.

While recognizing that MSS is a multisystem condition, in this review, we will specifically discuss MSS myopathy, thoroughly dissecting the pathological mechanisms and drawing comparisons with other disorders that share a similar phenotypic picture.

### 2.2. Skeletal Muscle Histopathology in MSS

The first reported histological analysis of MSS tissues described severe cortical ribbon atrophy in the cerebellum, primarily impacting the vermis rather than the lateral lobes. Very few Golgi nerve cells were left, and the Purkinje and granule cells had disappeared entirely. The cerebral hemispheres, which have properly myelinated white matter and no gliosis, showed no abnormalities. Histological analysis of the gastrocnemius revealed a reduction in the number and size of fibers, along with fatty accumulation between muscle fiber bundles [21]. These results showed the degeneration of muscle fibers, which were replaced by fatty tissue in both samples examined. This phenomenon is commonly recognized as a characteristic of declining muscle strength, architecture, contraction, and overall capacity [22].

Over the following 40 years, the analysis of numerous MSS muscle biopsy case reports at the microscopic and ultrastructural levels revealed similar features in most MSS patients despite variations in sex, age, ethnicity, and familial history. The main histological feature observed in MSS muscles is increased variability in muscle fiber size, with no predominance of one fiber type over the others. This peculiarity has been described in most of the case reports [12,19,23,24,25,26,27,28,29,30,31]. The increasing variability in fiber size, as well as fatty replacement, are standard features of atrophic and degenerating muscle fibers. Although fiber size variability is the most reported abnormality in MSS muscles, others have noted the predominance of a specific fiber type. As an example, two studies observed the preponderance of type 2C fibers [32,33]. In contrast, others reported an imbalance in type 2B fibers [34], the predominance of type 1 fibers over type 2 fibers [35,36], or simply the massive presence of type 1 fibers compared to the other types [37]. The different distribution of muscle fibers observed, with the predominance of those with slow or fast contraction, does not appear to be specifically associated with the clinical condition, given the high variability in the samples. Moreover, the fiber type predominance is not related to patients’ age. Indeed, these different features were observed in patients aged 18 months to 36 years.

Two other features highlighted in most MSS are the replacement of muscle fibers with amorphous material and adipose tissue [24,27,30,35,38,39], as well as the vacuolar degeneration [12,23,24,26,27,28,29,30,31,32,34,36,37,38,39].

### 2.3. Pathophysiological Mechanisms

In addition to “macroscopic” features, MSS muscles also exhibit peculiarities at the ultrastructural level. Changes in the size and structure of mitochondria were observed in the samples of seven patients aged 16 to 41 years [30]. One might speculate that this feature appears as the disease progresses. However, the high prevalence of aberrant mitochondria in two patients aged 18 months and 3 years suggests a possible early onset of this pathological phenotype in skeletal muscle [27]. Some studies reported a normal condition of mitochondrial shape and size [23,40] despite degeneration and internalization of myonuclei.

A substantial body of research has highlighted the presence of atypical material in the perinuclear space, characterized in various ways as thick [12,23], tight [32], and electron-dense [37,39]. These distinctive structures encircle the myonuclei [19,26], revealing a complex cellular environment. Moreover, they manifest in several intriguing forms, such as myeloid membrane-bound bodies [27], membrane-bound lipid vacuoles [30], membrane whorls [33], and membranous materials linked to autophagic or mitophagic processes [25]. While autophagy plays a beneficial role in controlling and regulating muscle mass, its complete absence may have an adverse impact on organelle shaping, leading to the accumulation of atypical mitochondria and dilated sarcoplasmic reticulum [41]. This compelling evidence highlights the intricate nature of cellular machinery and its significant implications for understanding cellular function and disease.

### 2.4. Animal Models and Translational Insights of MSS

Muscle biopsies are useful for examining the underlying causes of pathologies and better understanding the downstream processes that lead to myopathy. Although these tissue samples can provide valuable information, a preclinical model that mimics the clinical features of the disease is needed to study all potential therapeutic approaches and fully understand the underlying mechanisms.

There are currently two mice models of MSS that have a disruption in the mouse *Sil1* gene. The CxB5/ByJ genetic background of the woozy (*wz*) mouse model, was born from a spontaneous mutation of the *Sil1* gene. An ETn retrotransposon has been spontaneously inserted in the *Sil1* intron 7 of this murine model, bringing an in-frame stop codon 96 nucleotides following the retrotransposon insertion sequence [42]. Similarly, others used an in-frame β-geo gene-trap cassette placed after exon 7 in a C57BL/6J mouse to create the *Sil1*^Gt^ model [42]. The insertion of the β-geo portion into the construct replaces the remaining part, leading to the elongation of *Sil1*. Despite originating from different mouse models, both result in the loss of SIL1 function and display all the characteristics of MSS patients, offering new opportunities for studying the disease. Roos and colleagues published the first study utilizing these preclinical models, focusing on skeletal muscle [26]. They discovered that woozy mice exhibit a gradual myopathy that resembles the vacuolar and myonuclear features of human muscles. The appearance of mitochondrial alterations, the development of autophagic vacuoles, and anomalies in the nuclear envelope are characteristic of this myopathy. The authors propose that the main reason for the observed changes in the perinuclear space was a focal disconnection of the nuclear envelope from the nuclear lamina and the chromatin. Furthermore, they noted a marked increase in the UPR caused by ER stress [26]. Later, the same group thoroughly examined the molecular and cellular causes of reduced ER–chaperone function and its impact on the integrity of skeletal muscle and the neurological system [43]. They compared survival (BCL2-BCL2 Apoptosis Regulator) and death (Poly (ADP-Ribose) Polymerase 1-PARP1) markers between neocerebellar (lobules I–VIII) and vestibulocerebellar (lobules IX–X) Purkinje cells. Indeed, higher levels of PARP1 and lower levels of BCL2 were found in the neocerebellar area. This finding confirms that vestibulocerebellar PCs continue to grow and survive during the first year of life, while neocerebellar PCs degenerate completely within the first three months of life, as previously reported by Zhao and colleagues [42].

When the skeletal muscle of woozy mice was examined microscopically, vacuolar myopathy with various calibers of muscle fibers and non-subsarcolemmal myonuclei emerged. Transmission electron microscopy images of the quadriceps of 16-week-old woozy mice revealed ultrastructural changes that were not observed in wild-type (WT) animals [44]. Similarly to previous observations in MSS patients, these changes were characterized by enlargement of the sarcoplasmic reticulum in the myofibrillar compartment and the presence of perinuclear autophagic vacuoles containing myelin-like membranous material [12,26]. Another group observed the same change in 26-week-old woozy mice in the same tissue [25]. In addition to the accumulation of cytoskeletal elements resembling spheroid morphologies and membranous material, they also detected autophagic material in Schwann cells, as well as a significant reduction in the number of neuromuscular connections.

Remarkably, following treatment with GSK2606414, a protein kinase R (PKR)-like ER kinase (PERK) inhibitor that delays neurodegeneration and the development of motor dysfunction, muscle abnormalities were less evident [44], suggesting potential therapeutic strategies for human patients. Notably, heterozygous woozy mice do not exhibit any skeletal muscle alterations despite the monoallelic *Sil1* loss of function [45]. The finding that heterozygous mice do not exhibit a pathological phenotype validates the autosomal recessive inheritance pattern and reinforces the intriguing possibility for gene dosage effects.

Even though the two previously mentioned murine models of MSS differ genetically, the skeletal muscle of the *Sil1*^Gt^ mice exhibits the same traits. Indeed, the loss of Sil1 impacts several cellular organelles, pathways critical to muscle physiology, and the expression of several secretory pathway proteins synthesized in the ER [46].

Kawahara and Hayashi generated an MSS zebrafish model by injecting morpholino antisense oligos targeting the *sil1* gene into zebrafish eggs [47]. The *sil1* morphants showed abnormal skeletal muscle architecture and a decrease in Purkinje cells in the cerebellar region. These zebrafish also exhibited increased markers related to ER stress, autophagy, and apoptosis (BiP, LC3, activated caspase 3), consistent with results obtained in human and mouse models of MSS. Interestingly, these animals exhibited smaller eyes, suggesting a potential role of sil1 in eye development and possibly linking it to the development of cataracts that occur in MSS patients [47]. Using the same zebrafish model, another group observed impaired integrity of the neuromuscular connections due to loss of functional sil1 and validated the altered condition of skeletal muscle [25].

Both mouse models and human patients with MSS exhibit similar ultrastructural changes, including enlarged sarcoplasmic reticulum, perinuclear autophagic vacuoles, and the accumulation of membranous material. Additionally, the progressive myopathy observed in woozy mice reflects the vacuolar and myonuclear features found in human muscle biopsies. Mitochondrial changes are present in both species, indicating shared pathophysiological mechanisms. Research using mouse models has confirmed that SIL1 dysfunction triggers UPR activation and ER stress, supporting the proposed mechanism of the disease in humans. Furthermore, the selective pattern of Purkinje cell degeneration seen in mice—where neocerebellar cells degenerate within three months while vestibulocerebellar cells survive for a longer period—offers valuable insights into the timing and regional specificity of neurodegeneration.

## 3. Comparative Analysis of MSS with Other ARCAs or Myopathies

Unlike MSS, cerebellar ataxia generally affects the heart muscle; however, it can also cause skeletal muscle changes that are not well understood. Here, we highlight the common characteristics between MSS and the other ARCAs, summarizing the significant publications that report the histological investigation of muscle specimens in these diseases.

Among ARCAs, Friedreich’s Ataxia affects 2–4 persons per 100,000, making it the most common ataxia in terms of prevalence. The leading cause of FRDA is a triplet expansion (GAA) n in the intron of the frataxin gene (*FXN*) between exons 1 and 2, as well as deletions, insertions, and missense and nonsense mutations. Frataxin, a crucial mitochondrial protein involved in Fe-S cluster synthesis, is encoded by the *FXN* and exhibits its highest expression levels in tissues with elevated metabolic activity [48,49]. Alterations in this protein lead to an accumulation of iron in the mitochondrial area, which inhibits the respiratory chain and increases oxidative stress levels [50]. Patients with FRDA exhibit gait deficits and limb ataxia, abnormalities in eye movements, tendon areflexia, and loss of proprioceptive awareness in their lower limbs. They usually lose the ability to walk about 15 years after the first manifestation of the disease, although a magnetic resonance imaging (MRI) of the brain showed no abnormalities in the cerebellum. Sex may significantly influence the severity of cardiac disease in people with FRDA, with female patients exhibiting less severe conditions than their male counterparts [51].

A proteomic study of gastrocnemius muscle biopsies from five FRDA patients revealed 228 differentially expressed (DE) proteins compared to control subjects [52]. In particular, the discovered DE proteins interact with cytoskeleton, muscle-specific components, ribosomal biogenesis, and many other mitochondrial pathways. Although they did not detect iron accumulation, skeletal muscle from five FRDA patients exhibited decreased mitochondrial function (complexes II and III) and aconitase activity compared with healthy individuals [53]. The lack of frataxin could explain the reduced mitochondrial function, but it is insufficient to explain the altered aconitase activity. During aerobic activity, the delayed kinetics of phosphocreatine recoveries (PCr) further supported the hypothesis that FRDA patients have reduced mitochondrial function [54]. Since skeletal muscle is rich in mitochondria and depends on efficient oxidative energy metabolism, it is not surprising to observe this effect, even though it is not the primarily affected tissue in FRDA.

The biopsies of the gastrocnemius muscles of four FRDA patients showed modest, generic myopathic alterations with selective type 2 fiber atrophy and fiber type grouping [55]. In more detail, this muscle exhibited reduced activity of respiratory chain complexes I and IV, myofibers with a smaller cross-sectional area (CSA), mild morphological changes in mitochondria, and, in one patient, increased lipid content. This scenario suggests a quantitative reduction in mitochondria rather than a dysfunction [55]. Subsequently, the same group examined the oxidative energy metabolism of calf muscle in seven other FRDA patients and observed similar results. This suggests that skeletal muscle is involved in this type of ataxia, even in the absence of clinical signs of mitochondrial myopathy [56].

ARCA encompasses many classes of ataxias, which are less common than FRDA. SCAR8 or recessive ataxia, is triggered by mutations of the spectrin repeat containing nuclear envelope protein 1 (*SYNE1*) gene [57]. This gene encodes the protein Nesprin-1, which is widely expressed in striated muscles and the cerebellum [58]. In a case study, a homozygous mutation c.26236C > T that results in an early stop codon in the last exon of the *SYNE1* gene was examined using muscle biopsy analysis. The quadriceps ultrasound indicated the replacement of healthy muscle tissue by fibrosis and fatty infiltration. The authors also noted an expansion of the perinuclear space in muscle cells and variability in fiber size without an increase in the number of muscle fibers or centralization of nuclei. [59].

Type 2 atrophic fibers with few cytochrome c oxidase (COX) negative fibers were observed in the histological examination of muscle biopsies from two women with gradually developing pure cerebellar ataxia [60]. In this case, identifying two pathogenic mutations in the *SYNE1* gene allowed the clinical diagnosis of ARCA1 to be substituted for the original diagnosis of mitochondrial disease. An intriguing study by Lamperti and colleagues examined the muscle samples of 135 subjects with cerebellar atrophy and ataxia but no genetic diagnosis. Although muscle biopsies showed no abnormalities, they noticed reduced concentrations of CoQ10 in the muscular tissues of 13 patients, allowing for an early diagnosis of CoQ10 deficiency [61]. This further underscores the importance of analyzing muscles in diagnosing ataxias, considering the potential benefits of early CoQ10 supplementation in this disease.

Muscle biopsy is useful not only to verify or examine previously identified mechanisms of ataxias but also to investigate some disorders whose mechanisms affect both skeletal muscle and the cerebellum. In the case of progressive cerebellar ataxia, muscle biopsy is a useful diagnostic tool when patients are suspected of having a mitochondrial disease, as demonstrated by Bargiela and colleagues, who observed mitochondrial dysfunctions in 23% of muscle biopsies from patients with progressive cerebellar ataxia [62]. A muscle biopsy may help assess new mitochondrial DNA (mtDNA) variants in adult-onset mitochondrial disorders, classified in the mitochondrial recessive ataxia syndrome (MIRAS) category. For example, using muscle biopsy, independent studies identified novel mtDNA variants in the cytochrome C oxidase II (*MT-CO2*) gene, encoded at the mitochondrial level, in cases where extensive genetic screening had failed to determine the cause of progressive cerebellar syndrome with sensory neuropathy [63,64]. Similarly, after 20 years of progressive ataxia manifestation, muscle biopsy helped to identify MERRF (myoclonus epilepsy with ragged-red fibers) syndrome [65]. In this category we can include also the SPG7, a genetic spastic ataxia typically manifesting in adulthood (ages 10–72) with gradual bilateral lower limb weakness, spasticity, and occasionally severe cerebellar ataxia [66]. Muscle biopsies from SPG7 individuals showed variability in fiber size and evidence of mitochondrial respiratory chain dysfunction, including COX-negative muscle fibers [67,68,69,70], as well as alterations in vacuoles [71]; however, some samples showed no histological abnormalities [72,73].

Among ARCAs associated with impaired DNA repair mechanisms, ataxia-telangiectasia, also known as Louis–Bar syndrome, is the second most common childhood-onset neurodegenerative disorder after Friedreich’s ataxia, with an estimated prevalence of 1 in 40,000 to 1 in 100,000 live births [74]. The neurological manifestations of A–T include dysarthria, oculomotor apraxia, extrapyramidal symptoms, axonal neuropathy, and varying degrees of cognitive impairment.

Individuals with A–T may also present with immunological and endocrine abnormalities, and an increased risk of developing neoplastic diseases [75]. Furthermore, patients exhibit muscle weakness [76], likely resulting from muscle fibrosis or fatty infiltration, as evidenced by ultrasound imaging [77]. Weakness and impaired coordination of the respiratory muscles contribute to compromised ventilation in these patients [78,79]. Unfortunately, no studies involving muscle biopsies have been reported to date, which limits our understanding of the underlying muscular pathology.

In terms of oculomotor apraxia type 1 (AOA1), only a few histological studies have examined muscle pathology, with some reporting pronounced fiber-type grouping in the quadriceps [80]. In contrast, others found no significant alterations in muscle architecture [81]. Similarly, histological examination of the deltoid muscle in an AOA2 patient revealed no abnormalities compared to normal muscle tissue [82].

In the context of congenital ataxias, muscle biopsies have primarily been used to investigate potential mitochondrial abnormalities rather than general histological changes. In Joubert syndrome, muscle biopsy has been used to confirm the presence of mitochondrial dysfunction [83] and to identify histological differences between atrophied and hypertrophied fibers [84]. To date, no histological examinations have been performed in the muscles of individuals with Cayman ataxia.

Lastly, among the rare metabolic ataxias, ataxia with vitamin E deficiency (AVED) has long been associated with a clinical phenotype resembling FRDA, typically accompanied by markedly reduced serum vitamin E levels. Muscle biopsy from a 14.5-year-old patient with AVED revealed rare myofibrillar disorganization and mild, non-specific cytoarchitectural abnormalities, consistent with subtle myopathic changes [85]. Additionally, the biopsy revealed the presence of rimmed vacuoles, inflammatory infiltrates, and occasional necrotic fibers [86]. In Wilson’s disease, where muscle cramps and weakness occur, muscle biopsies have shown no abnormalities [87]. Currently, there are no reported histological examinations of muscle biopsies from individuals with Refsum’s disease in the literature.

All the details reported in the above discussed clinical studies are summarized in Table 1.

### ARCAs Animal Models

Preclinical models for FRDA are typically created using conditional FXN-MCK (muscle creatine kinase) mice, a tissue-specific *FXN*-knockout in cardiac and skeletal muscle [89]. According to Puccio and colleagues, this model mimics the increasing clinical and biochemical aspects of human disease; however, despite deleting frataxin in skeletal muscle, no histological, biochemical, or ultrastructural defects were observed [90]. These observations suggest that, despite the high frataxin levels in normal skeletal muscles, they are less susceptible to frataxin deficiency than the heart, as has already been observed in FRDA patients [91,92]. In a study evaluating (adeno-associated virus serotype 8) AAV8-mediated reintroduction of frataxin in FXN-MCK mice, Chang and colleagues found no microscopic changes in the quadriceps of the vehicle-treated animals [89].

Meanwhile, in another mouse model of inducible FXN depletion (TG), reduced muscle mass growth was shown, with smaller myofiber CSA, reduced translation of all proteins globally and no increase in autophagy. In the grip strength test, the TG mice exhibited lower forelimb and hindlimb strength than the WT mice. Additionally, the skeletal muscle of the same animals exhibited a considerable increase in phosphorylated eukaryotic initiation factor 2α subunit (p-eIF2α) protein levels [93]. Hui and colleagues, using a mouse model in which frataxin was temporarily suppressed by treatment with doxycycline, studied aconitase activity in the brain, cerebellum, and quadriceps before and after treatment with dimethyl fumarate (DMF) [94,95]. The quadriceps of mice deprived of frataxin significantly reduced mitochondrial function and overall protein expression. Interestingly, after 2 weeks of treatment with DMF, frataxin and cytochrome C levels partially recovered and then rose again, indicating the potential of DMF as a therapeutic option for FRDA. Although no histological examination was performed on skeletal muscle, mouse models of conditional FXN depletion exhibited poor coordination and muscle impairment in the notched-bar test [96], as well as in the accelerating rotarod [96,97,98], treadmill [99], and, in general, activity within their cage [100]. These motor issues were rescued after (Herpes simplex virus) HSV- or AAV-mediated FXN reintroduction.

While skeletal muscle shows less vulnerability to frataxin deficiency in preclinical models of Friedreich’s ataxia compared to cardiac muscle, this may not accurately represent how the disease progresses in humans. Some mouse models, such as the FXN-MCK mice, exhibit no histological defects in skeletal muscle despite the absence of the frataxin protein, emphasizing the existence of species-specific tissue vulnerabilities. Regarding other biological classes of ARCAs, the understanding of muscle phenotypes in available preclinical models remains limited. A zebrafish model was developed by knocking out the DNA polymerase gamma gene (*polg*) to study the effects associated in humans with mitochondrial recessive ataxia syndrome. Facchinello and colleagues found that both larval and adult *polg*^−/−^ zebrafish exhibited reduced mitochondrial mass, which resulted in altered muscle fibers [101]. Within the congenital ataxia, a *Bnip-h* (*Atcay*) knockdown zebrafish was developed to investigate the molecular mechanisms of Cayman syndrome. Abnormal structures were observed in both slow and fast muscle types using this model [102]. However, as previously mentioned, it remains challenging to draw comparisons with human patients, as no muscle analyses have been reported in the literature to date.

To date, no muscle histological investigations have been published for preclinical models of other ARCAs, including those involving impaired DNA repair mechanisms and metabolic ataxias.

All the details reported in the above-mentioned preclinical studies are summarized in Table 2.

## 4. Clinical and Therapeutic Implications

In our examination of cerebellar ataxias, particularly focusing on Marinesco–Sjögren Syndrome (MSS), muscle biopsy analysis has emerged as a crucial diagnostic tool. However, it is not the primary investigative approach. Muscle biopsy serves as an important technique for confirming or adjusting a diagnosis and determining the most appropriate treatment. However, muscle is not the first tissue typically examined when identifying cerebellar ataxias.

Among ARCAs, MSS displays a distinct clinical profile, characterized by severe skeletal muscle involvement, unlike other ARCAs, which primarily present with non-muscular issues. Another difference compared to other ARCA is the absence of sex contribution, which has instead been observed in FRDA [51], SCAs [103,104], Joubert syndrome [105], and Wilson’s disease [106,107,108]. Comparative analysis with other ARCAs, particularly FRDA, SCAR2, AOA1, Joubert syndrome, and AVED, has revealed notable similarities in muscle biopsy findings, including myopathic changes and fatty infiltration. However, distinctive autophagy patterns observed in muscle fibers represent a key differentiating feature of MSS compared to other biological classes of ARCAs (see Table 1 for details).

The development of murine models, particularly the woozy and *Sil1*^Gt^ variants, has significantly advanced our understanding of the pathological processes in skeletal muscle and cerebellum. These models have demonstrated histological characteristics that parallel those of human samples, including autophagic vacuoles and mitochondrial alterations. Perhaps most intriguingly, these preclinical models have revealed a previously unknown pattern of selective Purkinje cell degeneration, with cells in the vestibulocerebellum showing early deterioration. In contrast, neocerebellar Purkinje cells display extended survival. This fascinating observation requires careful consideration regarding its relevance to human pathology, given the condition’s rarity and limited tissue availability. These results enhance our understanding of MSS and guide targeted interventions on specific muscle regions in future medical efforts, as well as the importance of muscle tissue analysis in the context of cerebellar ataxias.

Alternative models, such as zebrafish, can complement mouse model studies by providing advantages in developmental timing, visualization capabilities, and screening potential. However, species differences, temporal differences, and genetic homogeneity highlight the importance of human tissue studies from early disease stages. These studies are crucial for validating therapeutic targets and understanding disease heterogeneity. Although this review does not address induced pluripotent stem cells (iPSCs), they represent a promising avenue for capturing human-specific disease mechanisms, particularly for the 40% of patients with MSS who do not have identified *SIL1* mutations. MSS iPSCs are especially valuable as they can differentiate into specific cell types affected by the disease, such as Purkinje and muscle cells, allowing researchers to study tissue-specific pathology. They also enable the investigation of disease progression from the earliest cellular changes and the testing of therapeutic compounds in a human cellular context. In conclusion, to fully understand the disorder and efficiently develop therapeutics, we must follow a multi-model approach, excluding the use of a single model system.

In summary, the examination of skeletal muscle holds significant value as a valuable tool within the realm of autosomal recessive cerebellar ataxias. Moving past the scope of MSS, the application of such investigations should be integrated into standard practice for additional pathologies falling under the umbrella of this disease category. A muscle biopsy is crucial for diagnosis, particularly due to the distinctive autophagy patterns that differentiate MSS from other ARCAs. The association between the onset of cerebellar and muscular symptoms highlight potential shared underlying mechanisms, which may improve diagnostic and therapeutic approaches. Consistent observations in muscle pathology across different patient populations suggest the potential for developing serum biomarkers that could aid in screening, diagnosis, and monitoring disease progression. This approach may help validate or improve the diagnosis of certain disorders, primarily when genetic testing alone does not provide comprehensive insights.

## Figures and Tables

**Table 1:** Patient Muscle Pathology Across Ataxic Syndromes. The symbol + indicates the finding in the category represented by the column. The double + indicates a medium modification, while the triple + indicates a massive modification.

	Observation						
	Fiber Size Variability	Fiber Type Grouping	Fatty Replacement	Mitochondrial Changes	Vacuolar Changes	Nuclear Changes	No Alterations
Ataxias with DNA repair defects							
A–T	-	-	-	-	-	-	-
AOA1/2	+ [80]	-	-	-	-	-	+ [81,82]
Degenerative ataxias							
FRDA	-	+ [55,56]	+ [55,56]	+ [55,56]	-	-	-
MSS	++ [12,21,23,24,26,27,28,30,32,33,34,35,36,37,38,39,40]	+ [33,34,35,36,37]	++ [21,24,27,30,32,34,35,37,38,39]	+++ [25,27,30,40]	+++ [12,23,25,26,28,30,31,32,34,35,36,37,38,39]	+++ [12,19,23,26,27,28,30,32,33,36,37,39]	-
CoQ10	-	-	-	-	-	-	+ [61]
ARCA-1 / SCAR8	+ [59]	-	+ [59]	-	-	+ [59]	-
SCAR2	+ [88]	-	-	-	-	+ [88]	-
MIRAS	-	+ [63,65]	-	++ [62,63,64,65]	-	-	-
SPG7	+ [68]	+ [67,68]	-	++ [67,68,69,70]	+ [71]	-	+ [72,73]
Congenital ataxias							
Joubert	-	-	+ [84]	+ [83]	-	-	+ [83]
Cayman	-	-	-	-	-	-	-
Metabolic ataxias							
AVED	+ [85,86]	-	-	-	-	+ [86]	-
Wilson’s	-	-	-	-	-	-	+ [87]
Refsum’s	-	-	-	-	-	-	-

**Table 2:** Muscle Pathology in Preclinical Models of Ataxias. ND: Not determined.

Disease	Preclinical Model (Name-Age)	Observations	Reference
Degenerative ataxias			
MSS	Woozy mouse - 26 to 100 weeks of age	− Muscle atrophy − Variation in fiber size − Internalized nuclei − Rimmed vacuoles − Autophagic structures − Membrane-bound vacuolar structures − Swollen mitochondria	[26]
MSS	Woozy mouse - 6 to 26 weeks of age	− Muscle atrophy − Autophagic vacuoles − Degenerating mitochondria − Degenerating nuclei	[43]
MSS	Woozy mouse - 16 weeks of age	− Enlargements of the sarcoplasmic reticulum − Perinuclear autophagic vacuoles containing myelin-like membranous material	[44]
MSS	Woozy mouse - 26 weeks of age	− Reduction in the size of NMJs	[25]
MSS	Woozy mouse - 5 to 71 weeks of age	− Atrophic fibers − Autophagic vacuoles	[45]
MSS	*Sil1*^Gt^ mouse - 3 to 15 months of age	− Variable fiber size − Internalized nuclei − Fatty replacement − Degenerating nuclei surrounded by membranous-bound structure	[46]
MSS	Morphant zebrafish - Embryos	− Markedly reduced normal patterns of birefringence − Disturbed myofibers formation	[47]
FRDA	FXN-MCK mice - 2 to 10 weeks of age	− No histological, biochemical, or ultrastructural defects	[90]
FRDA	Inducible *FXN* depletion (TG) mouse - 19 to 27 weeks of age	− Reduced growth of muscle mass, with smaller myofiber CSA, along with reduced translation of all proteins globally and no increase in autophagy	[93]
FRDA	Conditioned *FXN* depleted - 5.5 to 21.5 weeks of age	− Coordination and muscle impairment	[96]
MIRAS	*polg* knockout ENU zebrafish - Embryo and adults	− Altered fibers − Decrease in mitochondrial mass − Aberrant mitochondrial cristae	[101]
Ataxias with impaired DNA repair mechanism			
A–T		ND	
AOA1/2		ND	
Congenital ataxias			
Cayman ataxia	*atcay* knockdown zebrafish - adults	− Disrupted muscle development	[102]
Joubert syndrome		ND	
Metabolic ataxias			
AVED		ND	
Wilson’s disease		ND	
Refsum’s disease		ND	

## Supplementary Materials

The following supporting information can be downloaded at: https://www.mdpi.com/article/10.3390/ijms26146736/s1.

## Abbreviations

The following abbreviations are used in this manuscript:

AAV	Adeno-associate virus
ADCAs	Autosomal dominant cerebellar ataxias
AOA1/2	Oculomotor ataxia-apraxia type ½
ARCAs	Autosomal recessive cerebellar ataxias
ATF6	Activating transcription factor 6
A-T	Ataxia-telangiectasia
AVED	Ataxia with vitamin E deficiency
BCL2	BCL2 Apoptosis regulator
BiP/GRP78	ER chaperone immunoglobulin binding protein
BniP-h (Atcay)	Caytaxin
CSA	Cross-sectional area
COX	Cytochrome c oxidase
DE	Differentially expressed (proteins)
DMF	Dimethyl fumarate
ER	Endoplasmic reticulum
FRDA	Friedreich’s ataxia
FXN	Frataxia
HSV	Herpes simplex virus
iPSCs	Induced pluripotent stem cells
IRE-1	Inositol-requiring enzyme 1
LC3	Microtubule associated protein 1 light chain 3 Alpha (MAP1LC3A)
MCK	Muscle creatine kinase
MERRF	Myoclonus epilepsy with ragged-red fibers
MIRAS	Mitochondrial recessive ataxia syndrome
MRI	Magnetic resonance imaging
MSS	Marinesco–Sjögren syndrome
MT-CO2	mtDNA variant in the cytochrome C oxidase II
mtDNA	Mitochondrial DNA
NEF	Nucleotide exchange factor
ORP150/GRP170	150 kDa Oxygen-regulated protein
PARP1	Poly (ADP-Ribose) Polymerase 1
PERK	ER kinase
PCr	Phosphocreatine recoveries
PCs	Purkinje cells
PKR	Protein kinase R
Polg	DNA polymerase gamma
p-eIF2α	Phospho-eukaryotic initiation factor 2α
SCAR	Autosomal recessive spinocerebellar ataxia
SIL1	SIL1 nucleotide exchange factor
SPG7	Spastic paraplegic 7
SYNE1	Spectron repeat containing nuclear envelope protein 1
UPR	Unfolded protein response
WT	Wild-type
