# Supplementary material for: Skeletal Muscle Pathology in Autosomal Recessive Cerebellar Ataxias: Insights from Marinesco–Sjögren Syndrome
Source: Int J Mol Sci. 2025 Jul 14;26(14):6736. doi: 10.3390/ijms26146736 (PMC12295950; doi:10.3390/ijms26146736)
Supplement: Supplementary file 1 [file ijms-26-06736-s001.zip › ijms-3719913-supplementary.pdf]

Supplementary Table S1. Detailed clinical findings in ARCAs muscle biopsies.

Disorder	Patients<br>(N - age)	Findings	Reference
		Degenerative ataxias	
MSS	2<br>-<br>4 to 13 years old	– Generalized muscle wasting<br>– Severe hypotonia<br>– Reduction in number and size of fibers<br>– Fatty replacement	[1]
MSS	1<br>-<br>11 years old	– Generalized muscle weakness<br>– Hypotonia<br>– Variation in fibers size<br>– Fatty replacement<br>– Vacuolar degeneration with rimmed vacuoles<br>– Myeloid bodies and vacuoles beneath the sarcolemma or near the nucleus	[2]
MSS	6<br>-<br>9 months to 14 years old	– Hypotonia<br>– Atrophic muscle fibers<br>– Variation in fibers size<br>– Predominance of type 1 or type 2 fibres<br>– Nuclear inclusions<br>– Vacuolar degeneration	[3]
MSS	4<br>-<br>unspecified age	– Increased abnormally enlarged number of lysosomes, containing whorled lamellar or amorphous inclusion bodies	[4]
MSS	4<br>-<br>10 to 30 years old	– Hypotonia<br>– Variation in fibers size<br>– Fatty replacement<br>– Autophagic vacuoles, myeloid bodies in subsarcolemmal space	[5]
MSS	6<br>-<br>1.5 to 32 years old	– Degeneration in muscle fibers<br>– Variation in fibers size<br>– Fatty replacement<br>– Internalised nuclei<br>– Abnormal mitochondria	[6]
MSS	3<br>-<br>2 to 6 years old	– Variation in fibers size<br>– Dense membrane surrounding the nuclei<br>– Small vacuoles	[7]
MSS	12<br>-<br>1 to 36 years old	– Variation in fibers size<br>– Autophagic vacuoles and rimmed vacuoles<br>– Nuclear changes	[8]
MSS	1<br>-<br>33 months old	– Mild variation in fibers size<br>– Type 2B fibers deficiency<br>– Increase in fibers tissue<br>– Rimmed vacuole	[9]
MSS	3<br>-<br>26 to 31 years old	– Generalized muscle atrophy and weakness<br>– Marked variation in fibers size<br>– Predominance of type 1 fibers	[10]

		– Deficiency of type 2B fibers<br>– Fatty replacement<br>– Rimmed vacuoles	
MSS	7<br>-<br>2 to 36 years old	– Variation in fibers size<br>– Predominance of type 1 fibers<br>– Internalised nuclei<br>– Fatty replacement<br>– Autophagic vacuoles with myeloid body formation<br>– Massive necrosis with autophagic phenomenon	[11]
MSS	7<br>-<br>16 to 41 years old	– Loss of muscle fibers<br>– Variation in fibers size<br>– Fatty replacement<br>– Internalised nuclei<br>– Membrane-bound lipid vacuoles<br>– Abnormal mitochondria	[12]
MSS	1<br>-<br>2 years old	– Variation in fibers size<br>– Increased type 2C fibers<br>– Intermyofibrillar collections of membranous whorls and glycogen particles	[13]
MSS	8<br>-<br>1 to 38 years old	– Nuclei degeneration<br>– Membranous structures surrounding nuclei	[14]
MSS	2<br>-<br>5 and 6.5 years old	– Variation in fibers size<br>– Rare necrotic and regenerating fibers<br>– Membranous structures surrounding the nuclei<br>– Rimmed vacuoles	[15]
MSS	2<br>-<br>unspecified age	– Membranous autophagic/mitophagic material<br>– Vacuolar structures contained granular or membranous electron-dense material<br>– Aberrant, often widened endoplasmic reticulum structures	[16]
MSS	4<br>-<br>unspecified age	– Normal variation of fibers size<br>– Fibers necrosis<br>– Defect of mitochondria	[17]
MSS	9<br>-<br>22 to 58 years old	– Hypotonia<br>– Fatty and connective tissue replacement	[18]
MSS	4<br>-<br>unspecified age	– Variation in fibers size<br>– Atrophic fibers<br>– Fatty replacement<br>– Autophagic vacuoles associated with degenerating myonuclei<br>– Internalised nuclei surrounded by membranous structures	[19]
MSS	3	– Atrophic fibers	[20]

	- 23 to 31 years old	– Degenerating nuclei surrounded by membranous structures – Autophagic vacuoles – Glycogen accumulation	
FRDA	4 - unspecified age	– Type 2 fibers atrophy – Fibers type grouping – Reduced MSA in myofibers – Fatty replacement – Aberrant mitochondria	[21]
FRDA	7 - unspecified age	– Type 2 fibers atrophy – Fibers type grouping – Reduced MSA in fibers – Fatty replacement – Aberrant mitochondria	[22]
ARCA-1	1 - unspecified age	– Variation in fibers size – Fatty and fibrous tissue replacement – Enlarged perinuclear space	[23]
SCAR2	1 - 4 years old	– Atrophic fibers – Internalised nuclei	[24]
Cerebellar ataxia with CoQ10 deficiency	13 - 6 to 35 years old	– No histological alterations	[25]
MIRAS	29 - Mean of 52 years old	– Mitochondrial dysfunctions	[26]
MIRAS	1 - 45 years old	– COX deficiency – Ragged-red fibres	[27]
MIRAS	1 - 52 years old	– COX deficiency	[28]
MIRAS	1 - 66 years old	– Increased subsarcolemmal mitochondrial proliferation – Ragged-red fibers	[29]
SPG7	1 - unspecified age	– No histological alterations	[30]
SPG7	1 - unspecified age	– Increased fat in vacuoles	[31]
SPG7	1 - 54 years old	– No histological alterations	[32]
SPG7	1 - 51 years old	– Increased type 2C fibers – COX defient fibers	[33]
SPG7	1 - 70 years old	– Variability in fiber size – Type 2 fibers atrophy – COX deficient fibers	[34]

SPG7	2<br>-<br>Unspecified age	– Ragged red fibers<br>– COX deficient fibers	[35]
SPG7	1<br>-<br>Unspecified age	– Ragged red fibers<br>– COX deficient fibers	[36]
Ataxias with impaired DNA repair mechanism			
A-T		**ND**	
AOA1	1<br>-<br>67 years old	– Variation in fibers size	[37]
AOA1	1<br>-<br>8 years old	– No alterations	[38]
AOA2	1<br>-<br>37 years old	– No histological alterations	[39]
Congenital ataxias			
Cayman ataxia		**ND**	
Joubert Syndrome	1<br>-<br>44 months	– No histological alterations<br>– Decreased pyruvate oxidation rates<br>– Decreased ATP production	[40]
Joubert Syndrome	2<br>-<br>12 and 15 years old	– Fatty and fibrous tissue replacement	[41]
Metabolic ataxias			
AVED	1<br>-<br>14.5 years old	– Myofibrillar derangement<br>– Mild, non-specific cytoarchitectural abnormalities<br>– Mild myopathic changes	[42]
AVED	1<br>-<br>69 years old	– Endomysial mononuclear inflammatory infiltrates<br>– Rimmed vacuoles;	[43]
Wilson's disease	1<br>-<br>10 years old	– No histological alterations	[44]
Refsum's disease		**ND**	

References

1. MAHLOUDJI, M., AMIRHAKIMI, G. H., HAGHIGHI, P., & KHODADOUST, A. A. (1972). MARINESCO-SJÖGREN SYNDROME REPORT OF AN AUTOPSY. *Brain*, *95*(4), 675–680. https://doi.org/10.1093/brain/95.4.675
2. Sasaki, K., Suga, K., Tsugawa, S., Sakuma, K., Tachi, N., Chiba, S., & Imamura, S. (1996). Muscle pathology in Marinesco-Sjo¨gren syndrome: a unique ultrastructural feature. *Brain and Development*, *18*(1), 64–67. https://doi.org/10.1016/0387-7604(95)00088-7
3. Zimmer, C., Gosztonyi, G., Cervos-Navarro, J., von Moers, A., & Schröder, J. M. (1992). Neuropathy with lysosomal changes in Marinesco-Sjögren syndrome: fine structural findings in skeletal muscle and conjunctiva. *Neuropediatrics*, *23*(6), 329–335. https://doi.org/10.1055/s-2008-1071368
4. Walker, P. D., Blitzer, M. G., & Shapira, E. (1985). Marinesco-Sjögren syndrome. *Neurology*, *35*(3), 415–415. https://doi.org/10.1212/WNL.35.3.415
5. Herva, R., von Wendt, L., von Wendt, G., Saukkonen, A. L., Leisti, J., & Dubowitz, V. (1987). A syndrome with juvenile cataract, cerebellar atrophy, mental retardation and myopathy. *Neuropediatrics*, *18*(3), 164–169. https://doi.org/10.1055/s-2008-1052473
6. Superneau, D. W., Wertelecki, W., Zellweger, H., & Bastian, F. (1987). Myopathy in Marinesco-Sjogren syndrome. *European Neurology*, *26*(1), 8–16. https://doi.org/10.1159/000116305
7. Sewry, C. A., Voit, T., & Dubowitz, V. (1988). Myopathy with unique ultrastructural feature in Marinesco-Sjögren syndrome. *Annals of Neurology*, *24*(4), 576–580. https://doi.org/10.1002/ana.410240416
8. Suzuki, Y., Murakami, N., Goto, Y., Orimo, S., Komiyama, A., Kuroiwa, Y., & Nonaka, I. (1997). Apoptotic nuclear degeneration in Marinesco-Sjögren syndrome. *Acta Neuropathologica*, *94*(5), 410–415. https://doi.org/10.1007/s004010050727
9. Ishikawa, T., Kitoh, H., Awaya, A., & Nonaka, I. (1993). Rapid cataract formation in Marinesco-Sjögren syndrome. *Pediatric Neurology*, *9*(5), 407–408. https://doi.org/10.1016/0887-8994(93)90114-r
10. Komiyama, A., Nonaka, I., & Hirayama, K. (1989). Muscle pathology in Marinesco-Sjögren syndrome. *Journal of the Neurological Sciences*, *89*(1), 103–113. https://doi.org/10.1016/0022-510x(89)90010-5
11. Goto, Y., Komiyama, A., Tanabe, Y., Katafuchi, Y., Ohtaki, E., & Nonaka, I. (1990). Myopathy in Marinesco-Sjögren syndrome: an ultrastructural study. *Acta Neuropathologica*, *80*(2), 123–128. https://doi.org/10.1007/BF00308914

12. Torbergsen, T., Stålberg, E., Aasly, J., & Lindal, S. (1991). Myopathy in Marinesco-Sjögren syndrome: an electrophysiological study. *Acta Neurologica Scandinavica*, *84*(2), 132–138. https://doi.org/10.1111/j.1600-0404.1991.tb04921.x

13. Tachi, N., Nagata, N., Wakai, S., & Chiba, S. (1991). Congenital muscular dystrophy in Marinesco-Sjögren syndrome. *Pediatric Neurology*, *7*(4), 296–298. https://doi.org/10.1016/0887-8994(91)90049-Q

14. Senderek, J., Krieger, M., Stendel, C., Bergmann, C., Moser, M., Breitbach-Faller, N., … Zerres, K. (2005). Mutations in SIL1 cause Marinesco-Sjögren syndrome, a cerebellar ataxia with cataract and myopathy. *Nature Genetics*, *37*(12), 1312–1314. https://doi.org/10.1038/ng1678

15. Lagier-Tourenne, C., Chaigne, D., Gong, J., Flori, J., Mohr, M., Ruh, D., … Dollfus, H. (2002). Linkage to 18qter differentiates two clinically overlapping syndromes: congenital cataracts-facial dysmorphism-neuropathy (CCFDN) syndrome and Marinesco-Sjögren syndrome. *Journal of Medical Genetics*, *39*(11), 838–843. https://doi.org/10.1136/jmg.39.11.838

16. Phan, V., Cox, D., Cipriani, S., Spendiff, S., Buchkremer, S., O'Connor, E., … Roos, A. (2019). SIL1 deficiency causes degenerative changes of peripheral nerves and neuromuscular junctions in fish, mice and human. *Neurobiology of Disease*, *124*, 218–229. https://doi.org/10.1016/j.nbd.2018.11.019

17. Müller-Felber, W., Zafiriou, D., Scheck, R., Pätzke, I., Toepfer, M., Pongratz, D. E., & Walther, U. (2007). Marinesco Sjögren Syndrome with Rhabdomyolysis. A New Subtype of the Disease. *Neuropediatrics*, *29*, 97–101. https://doi.org/10.1055/s-2007-973542

18. Mahjneh, I., Anttonen, A.-K., Somer, M., Paetau, A., Lehesjoki, A.-E., Somer, H., & Udd, B. (2006). Myopathy is a prominent featurein Marinesco-Sjögren syndrome. *Journal of Neurology*, *253*(3), 301–306. https://doi.org/10.1007/s00415-005-0983-9

19. Krieger, M., Roos, A., Stendel, C., Claeys, K. G., Sonmez, F. M., Baudis, M., … Senderek, J. (2013). SIL1 mutations and clinical spectrum in patients with Marinesco-Sjogren syndrome. *Brain: A Journal of Neurology*, *136*(Pt 12), 3634–3644. https://doi.org/10.1093/brain/awt283

20. Roos, A., Buchkremer, S., Kollipara, L., Labisch, T., Gatz, C., Zitzelsberger, M., … Weis, J. (2014). Myopathy in Marinesco–Sjögren syndrome links endoplasmic reticulum chaperone dysfunction to nuclear envelope pathology. *Acta Neuropathologica*, *127*(5), 761–777. https://doi.org/10.1007/s00401-013-1224-4

21. Nachbauer, W., Boesch, S., Reindl, M., Eigentler, A., Hufler, K., Poewe, W., … Wanschitz, J. (2012). Skeletal Muscle Involvement in Friedreich Ataxia and Potential Effects of Recombinant Human Erythropoietin Administration on Muscle Regeneration and Neovascularization. *Journal of Neuropathology & Experimental Neurology*, *71*(8), 708–715. https://doi.org/10.1097/NEN.0b013e31825fed76

22. Nachbauer, W., Boesch, S., Schneider, R., Eigentler, A., Wanschitz, J., Poewe, W., & Schocke, M. (2013). Bioenergetics of the Calf Muscle in Friedreich Ataxia Patients Measured by 31P-MRS Before and After Treatment with Recombinant Human Erythropoietin. *PLoS ONE*, *8*(7), e69229. https://doi.org/10.1371/journal.pone.0069229

23. Baumann, M., Steichen-Gersdorf, E., Krabichler, B., Petersen, B.-S., Weber, U., Schmidt, W. M., … Janecke, A. R. (2017). Homozygous SYNE1 mutation causes congenital onset of muscular weakness with distal arthrogryposis: a genotype–phenotype correlation. *European Journal of Human Genetics*, *25*(2), 262–266. https://doi.org/10.1038/ejhg.2016.144

24. Jobling, R. K., Assoum, M., Gakh, O., Blaser, S., Raiman, J. A., Mignot, C., … Yoon, G. (2015). PMPCA mutations cause abnormal mitochondrial protein processing in patients with non-progressive cerebellar ataxia. *Brain*, *138*(6), 1505–1517. https://doi.org/10.1093/brain/awv057

25. Lamperti, C., Naini, A., Hirano, M., De Vivo, D. C., Bertini, E., Servidei, S., … DiMauro, S. (2003). Cerebellar ataxia and coenzyme Q10 deficiency. *Neurology*, *60*(7), 1206–1208. https://doi.org/10.1212/01.WNL.0000055089.39373.FC

26. Bargiela, D., Shanmugarajah, P., Lo, C., Blakely, E. L., Taylor, R. W., Horvath, R., … Hadjivassiliou, M. (2015). Mitochondrial pathology in progressive cerebellar ataxia. *Cerebellum & Ataxias*, *2*, 16. https://doi.org/10.1186/s40673-015-0035-x

27. Baty, K., Farrugia, M. E., Hopton, S., Falkous, G., Schaefer, A. M., Stewart, W., … Ng, Y. S. (2021). A novel MT-CO2 variant causing cerebellar ataxia and neuropathy: The role of muscle biopsy in diagnosis and defining pathogenicity. *Neuromuscular Disorders*, *31*(11), 1186–1193. https://doi.org/10.1016/j.nmd.2021.05.014

28. Zierz, C. M., Baty, K., Blakely, E. L., Hopton, S., Falkous, G., Schaefer, A. M., … Taylor, R. W. (2019). A Novel Pathogenic Variant in MT-CO2 Causes an Isolated Mitochondrial Complex IV Deficiency and Late-Onset Cerebellar Ataxia. *Journal of Clinical Medicine*, *8*(6), 789. https://doi.org/10.3390/jcm8060789

29. Pedroso, J. L., de Rezende Pinto, W. B. V., Barsottini, O. G. P., & Oliveira, A. S. B. (2020). Should we investigate mitochondrial disorders in progressive adult-onset undetermined ataxias? *Cerebellum & Ataxias*, *7*, 13. https://doi.org/10.1186/s40673-020-00122-0

30. Wilkinson, P. A., Crosby, A. H., Turner, C., Bradley, L. J., Ginsberg, L., Wood, N. W., … Warner, T. T. (2004). A clinical, genetic and biochemical study of SPG7 mutations in hereditary spastic paraplegia. *Brain*, *127*(5), 973–980. https://doi.org/10.1093/brain/awh125

31. Wedding, I. M., Koht, J., Tran, G. T., Misceo, D., Selmer, K. K., Holmgren, A., … Tzoulis, C. (2014). Spastic Paraplegia Type 7 Is Associated with Multiple Mitochondrial DNA Deletions. *PLOS ONE*, *9*(1), e86340. https://doi.org/10.1371/journal.pone.0086340

32. Mahoney, C. J., Dharmadasa, T., Huynh, W., Halpern, J.-P., Vucic, S., Mowat, D., & Kiernan, M. C. (2020). A novel phenotype of hereditary spastic paraplegia type 7 associated with a compound heterozygous mutation in paraplegin. *Muscle & Nerve*, *62*(1), E44–E45. https://doi.org/10.1002/mus.26882

33. McDermott, C. J., Dayaratne, R. K., Tomkins, J., Lusher, M. E., Lindsey, J. C., Johnson, M. A., … Shaw, P. J. (2001). Paraplegin gene analysis in hereditary spastic paraparesis (HSP) pedigrees in northeast England. *Neurology*, *56*(4), 467–471. https://doi.org/10.1212/wnl.56.4.467

34. van Gassen, K. L. I., van der Heijden, C. D. C. C., de Bot, S. T., den Dunnen, W. F. A., van den Berg, L. H., Verschuuren-Bemelmans, C. C., … van de Warrenburg, B. P. (2012). Genotype-phenotype correlations in spastic paraplegia type 7: a study in a large Dutch cohort. *Brain: A Journal of Neurology*, *135*(Pt 10), 2994–3004. https://doi.org/10.1093/brain/aws224

35. Casari, G., De Fusco, M., Ciarmatori, S., Zeviani, M., Mora, M., Fernandez, P., … Ballabio, A. (1998). Spastic paraplegia and OXPHOS impairment caused by mutations in paraplegin, a nuclear-encoded mitochondrial metalloprotease. *Cell*, *93*(6), 973–983. https://doi.org/10.1016/s0092-8674(00)81203-9

36. Arnoldi, A., Tonelli, A., Crippa, F., Villani, G., Pacelli, C., Sironi, M., … Bassi, M. T. (2008). A clinical, genetic, and biochemical characterization of SPG7 mutations in a large cohort of patients with hereditary spastic paraplegia. *Human Mutation*, *29*(4), 522–531. https://doi.org/10.1002/humu.20682

37. Nakamura, K., Yoshida, K., Makishita, H., Kitamura, E., Hashimoto, S., & Ikeda, S. (2009). A novel nonsense mutation in a Japanese family with ataxia with oculomotor apraxia type 2 (AOA2). *Journal of Human Genetics*, *54*(12), 746–748. https://doi.org/10.1038/jhg.2009.104

38. Shah, A., Byrd, P. J., Malcolm, A., Nestor, T., Ffr-Rcsi, S. R., & King, M. D. (2006). Atypical presentation of ataxia-oculomotor apraxia type 1. *Developmental Medicine & Child Neurology*, *48*(6), 529–532. https://doi.org/10.1111/j.1469-8749.2006.tb01308.x

39. Anheim, M., Fleury, M.-C., Franques, J., Moreira, M.-C., Delaunoy, J.-P., Stoppa-Lyonnet, D., … Tranchant, C. (2008). Clinical and Molecular Findings of Ataxia With Oculomotor Apraxia Type 2 in 4 Families. *Archives of Neurology*, *65*(7), 958–962. https://doi.org/10.1001/archneur.65.7.958

40. Morava, É., Dinopoulos, A., Kroes, H. Y., Rodenburg, R. J. T., Van Bokhoven, H., Van Den Heuvel, L. P., & Smeitink, J. A. M. (2005). Mitochondrial Dysfunction in a Patient with Joubert Syndrome. *Neuropediatrics*, *36*(3), 214–217. https://doi.org/10.1055/s-2005-865610

41. Arancio, O., Bongiovanni, L. G., Bonadonna, G., Tomelleri, G., & De Grandis, D. (1988). Congenital muscular dystrophy and cerebellar vermis agenesis in two brothers. *The Italian Journal of Neurological Sciences*, *9*(5), 485–489. https://doi.org/10.1007/BF02337167

42. Becker, A. E., Vargas, W., & Pearson, T. S. (2016). Ataxia with Vitamin E Deficiency May Present with Cervical Dystonia. *Tremor and Other Hyperkinetic Movements*, *6*(0). https://doi.org/10.5334/tohm.298

43. Kleopa, K. A., Kyriacou, K., Zamba-Papanicolaou, E., & Kyriakides, T. (2005). Reversible inflammatory and vacuolar myopathy with vitamin E deficiency in celiac disease. *Muscle & Nerve*, *31*(2), 260–265. https://doi.org/10.1002/mus.20144

44. Rosen, J. M., Kuntz, N., Melin-Aldana, H., & Bass, L. M. (2013). Spasmodic Muscle Cramps and Weakness as Presenting Symptoms in Wilson Disease. *Pediatrics*, *132*(4), e1039–e1042. https://doi.org/10.1542/peds.2012-2923
